# Supplementary material for: Ergosterol-depleted clinical isolates of Nakaseomyces glabratus can develop multi-drug resistance without apparent fitness and virulence defects
Source: bioRxiv. 2025 Aug 28:2025.08.28.672802. Preprint. [Version 1] doi: 10.1101/2025.08.28.672802 (PMC12407835; doi:10.1101/2025.08.28.672802)
Supplement: Supplement 5 [file NIHPP2025.08.28.672802v1-supplement-5.pdf]

Figure S1. Cell wall composition of MDR *N. glabratus* isolates.

The indicated *N. glabratus* strains were grown in YPD at 37°C, harvested and then stained for chitin, ß-1,3-D-glucan and mannan. The amounts of the three cell wall components were measured by flow cytometry. The values were normalized against the values of the control strain CBS138.

Figure S1

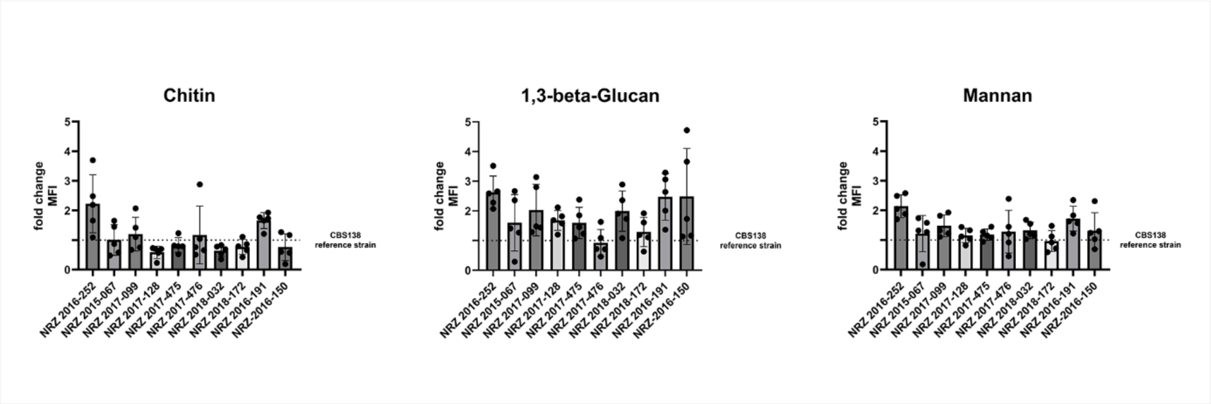
Chitin
1,3-beta-Glucan
Mannan
fold change MFI
CBS138 reference strain
NRZ 2016-252
NRZ 2015-067
NRZ 2017-099
NRZ 2017-128
NRZ 2017-475
NRZ 2017-476
NRZ 2018-032
NRZ 2018-172
NRZ 2016-191
NRZ-2016-150
